# Low-dose bitter leaf improves sperm quality disrupted in immunosuppressed Wistar rats: An experimental study

**DOI:** 10.18502/ijrm.v19i6.9379

**Published:** 2021-07-27

**Authors:** Mohsen Akbaribazm, Mozafar Khazaei

**Affiliations:** Fertility and Infertility Research Center, Health Technology Institute, Kermanshah University of Medical Sciences, Kermanshah, Iran.

##  Dear Editor

Recently, we read the valuable article entitled “Low dose Bitter leaf improves sperm quality disrupted in immunosuppressed Wistar rats: An experimental study” with interest that has been published online in International Journal of Reproductive Biomedicine (2020) (1) that evaluated the positive fertility effects of Bitter leaf through biochemical, hematological, and histopathological evidences. The work is incredible, however, among the most important findings here are a few comments that the authors should have considered in their article:

1) One of the important factors in analyzing the results of sperm and fertility parameters of the male sex is to measure the sex hormones such as testosterone, luteinizing hormone, and follicle-stimulating hormone of the intervention groups. Changes in the levels of these hormones can be an important indicator in determining the involvement of the treatment agent in the hypothalamic–pituitary–gonadal axis and changes in the level of activity of enzymes involved in the synthesis of testosterone (or dihydrotestosterone). Measuring the levels of these hormones for this study could help with the results (2, 3).

2) First, it was better to use hydroalcoholic extracts because water or alcohol alone can separate single-phase compounds. Many of the hydrophobic compounds do not come out of the plant powder during the preparation of aqueous extract and do not enter the final extract, and vice versa for alcoholic extracts. Second, it would be better to use the liquid chromatography electrospray ionization tandem mass spectrometric technique using M/Z (M-H${}^{-/+}$) to identify effective compounds. Because by analyzing the compounds in the extract, it is possible to understand the pathways associated with spermatogenesis process, mentioned in the discussion section of the article; there is no explanation about the testicular protective and aphrodisiac compounds which are present in this plant. Also, scanning electron microscopy/energy dispersive X-ray spectroscopy and inductively coupled plasma mass spectrometry techniques are also used to identify mineral elements. If heavy metals such as lead (Pb), mercury (Hg), and arsenic (As) or even important mineral elements involved in immunological pathways such as selenium (Se), cobalt (Co), copper (Cu), etc. are detected, changes in testis or semen function can also be attributed to these elements (4, 5).

3) In animal models and humans reactive oxygen species (ROS) by mediating cellular signal transduction pathways play a major role in testicular tissue and spermatogenesis process via lipid peroxidation (loss of membrane integrity), decrease in sperm motility, increase in abnormalities in spermatogenesis, increase in deoxyribonucleic acid damage, induced apoptosis, and finally initiating an ROS-related inflammatory reaction. Extracts with different polyphenolic compounds have the ability to change these active species. Various tissue and serum techniques, including malondialdehyde and nitric oxide levels, total antioxidant capacity, as well as glutathione peroxidase superoxide dismutase and catalase activity, can help with these findings and identify the pathways of ROS-mediated apoptosis pathways damages in testis paranchymal tissue (5).

4) Serum and tissue measurements of pro-inflammatory [interleukin-1 (IL-1), tumor necrosis factor alpha, interferon gamma, IL-12, IL-18 and granulocyte-macrophage colony-stimulating factor] and anti-inflammatory (IL-4, IL-10, IL-13, and interferon alpha) cytokines can also be used to evaluate the inflammatory pathways involved in the toxic or protective effect of bitter leaf extract (6).
